# Supplementary figures and images for: Metabolic disorders and post-acute hospitalization in black/mixed-race patients with long COVID in Brazil: A cross-sectional analysis
Source: PLoS One. 2022 Oct 31;17(10):e0276771. doi: 10.1371/journal.pone.0276771 (PMC9621406; doi:10.1371/journal.pone.0276771)

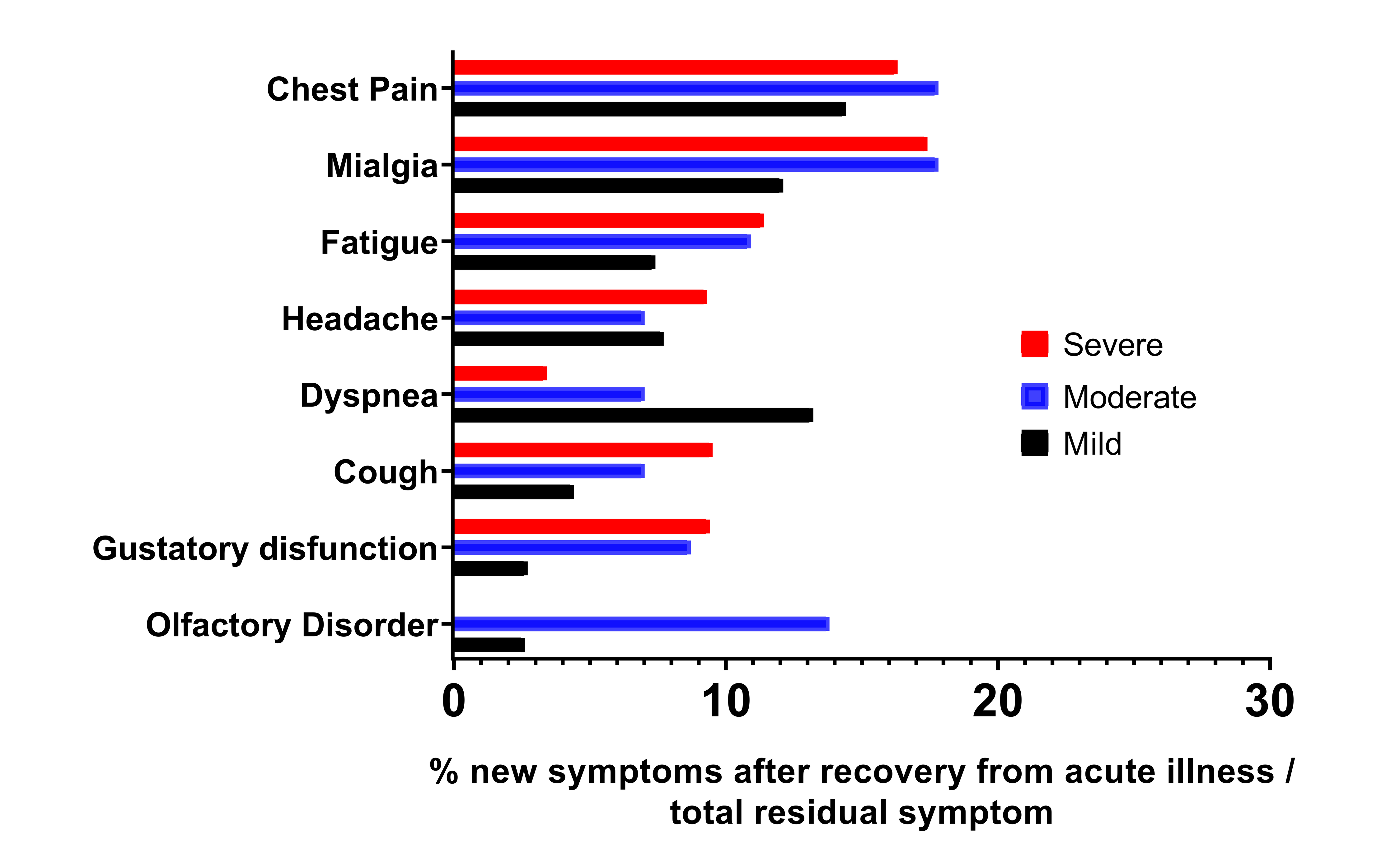

Supplement: S1 Fig — (TIF) [file pone.0276771.s001.tif]

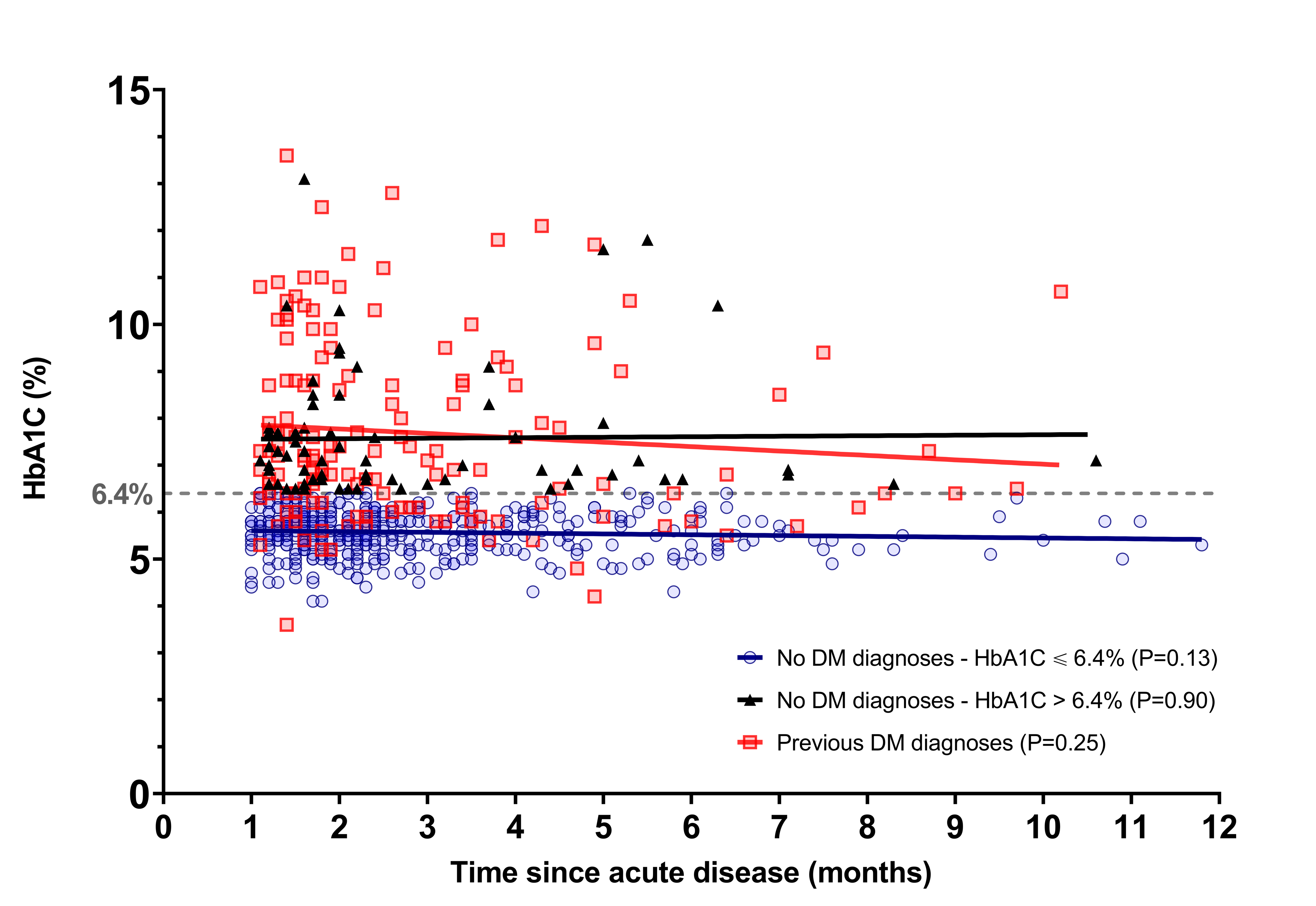

Supplement: S2 Fig — Patients with no previous diagnoses of DM were categorized according to test levels as ≤6.4% and >6.4%. Red squares are HbA1C levels of patients with previous diagnoses of DM, blue circles, no previous diagnoses of DM—HbA1C≤6.4%, and black triangles, no previous diagnoses of DM—HbA1C>6.4%. Red, blue, and black lines represent linear regression; significance levels are displayed in the graph legend. (TIF) [file pone.0276771.s002.tif]
