# Supplementary material for: Metabolic disorders and post-acute hospitalization in black/mixed-race patients with long COVID in Brazil: A cross-sectional analysis
Source: PLoS One. 2022 Oct 31;17(10):e0276771. doi: 10.1371/journal.pone.0276771 (PMC9621406; doi:10.1371/journal.pone.0276771)
Supplement: S1 Table — Data are n (%), n/N (%), mean (±SD) or median (IQR). (PDF) [file pone.0276771.s003.pdf]

Supplementary Table 1 – Acute phase symptoms of long COVID patients.

	n/N (%)
**Number of symptoms**	8.0 (6.0 - 10.0)
**Fever**	514/808 (63.6)
**Cough**	633/809 (78.2)
**Myalgia**	583/807 (72.2)
**Headache**	565/809 (69.8)
**Diarrhea**	329/809 (40.7)
**Dyspnea**	672/809 (83.1)
**Vomiting/Nausea**	293/809 (36.2)
**Olfactory disfunction**	473/808 (58.5)
**Gustatory disfunction**	475/809 (58.7)
**Fatigue/Muscle weakness**	668/809 (82.6)
**Chest pain**	514/809 (63.5)

Data are n (%), n/N (%), mean (±SD) or median (IQR)
