# Supplementary material for: Metabolic disorders and post-acute hospitalization in black/mixed-race patients with long COVID in Brazil: A cross-sectional analysis
Source: PLoS One. 2022 Oct 31;17(10):e0276771. doi: 10.1371/journal.pone.0276771 (PMC9621406; doi:10.1371/journal.pone.0276771)
Supplement: S2 Table — Data are described as n(%), n/N (%), or mead (±SD). Variant Period: 1- August 2020 to January 2021. 2- March 2021 to July 2021 [1]. (PDF) [file pone.0276771.s004.pdf]

Supplementary Table 02- Clinical presentation by calendar time according to variant predominance distribution.

	Ancestral [1] N=736	Gama-Variant[2] N=249
**Female Sex**	440/736 (59.8)	117/249 (47.0)
**Age**	52.1 (±14.2)	52.1 (±11.9)
**BMI**	29.7 (6.2)	30.3 (6.3)
**Any Comorbidities**	527 (71.6)	187/248 (75.4)
**Hospitalization**	443 (60.2)	236 (94.8)
ICU	236/443 (53.3)	167/236 (70.7)
**Headache**	243/685 (35.5)	83/248 (33.5)
**Cough**	269/735 (36.6)	106 (42.6)
**Dyspnea**	486 (66.0)	165 (66.3)
**Myalgia**	273/735 (37.1)	106 (42.6)
**Fatigue**	466/735 (63.4)	140 (56.2)
**Chest Pain**	337/734 (45.9)	95 (38.2)
**Appetite Loss**	121/734 (16.5)	33/237 (13.9)
**Dysphagia**	36/734 (4.9)	9/233 (3.9)
**Dysphonia**	39/734 (5.3)	12/234 (5.1)
**Gustatory disfunction**	124/622 (19.9)	17/233 (7.3)
**Smell Loss**	130/620 (21.0)	23/232 (9.9)
**Motor limitation**	107/619 (17.3)	52/235 (22.1)
**Hair Loss**	65/181 (35.9)	97/238 (40.8)
**Dizziness**	70/181 (38.7)	71/244 (29.1)
**Insomnia**	96/181 (53.0)	122/245 (49.8)
**Memory loss**	100/182 (54.9)	136/244 (55.7)

Data are described as n(%), n/N (%), or mead (±SD).
Variant Period: 1- August 2020 to January 2021. 2- March 2021 to July 2021 [1]

REFERENCE:

1. Katikireddi SV, Cerqueira-Silva T, Vasileiou E, Robertson C, Amele S, Pan J, et al. Two-dose ChAdOx1 nCoV-19 vaccine protection against COVID-19 hospital admissions and deaths over time: a retrospective, population-based cohort study in Scotland and Brazil. Lancet. 2022;399: 25–35. doi:10.1016/S0140-6736(21)02754-9
