# Supplementary material for: Metabolic disorders and post-acute hospitalization in black/mixed-race patients with long COVID in Brazil: A cross-sectional analysis
Source: PLoS One. 2022 Oct 31;17(10):e0276771. doi: 10.1371/journal.pone.0276771 (PMC9621406; doi:10.1371/journal.pone.0276771)
Supplement: S3 Table — Characteristics were chosen according to the potential to modulate HbA1C level. Group of patients were divided according to the level of HA1c. Data are in n (%) or n/N (%), in case of missing data. Only patients that presented hbA1C results were described. P-value <0.05 are highlighted. 1- “Corticosteroid use” refers to specific use at acute phase of disease. 2- Diagnostic based on the level of hemoglobin measured at first visit. 3- Full list of comorbidities is described in Table 1. 4- Newly diagnoses of DM. (PDF) [file pone.0276771.s005.pdf]

Supplementary Table 3 – Clinical characteristics of patients with and without previous diagnosis of DM. Characteristics were chosen according to the potential to modulate HbA1c level. Group of patients were divided according to the level of HA1c.

	PREVIOUS DIAGNOSES OF DM					
	No			Yes		
**HbA1c**	<6.4% N=441	>6.4% N=71	P-Value	<6.4% N=47	>6.4% N=104	P-Value
**Time since disease onset**	2.9 (±1.9)	2.9 (±1.9)	0.697	3.2 (±2.0)	2.6 (±1.8)	0.352
**Corticosteroid use**[1]	210/284 (74)	35/44 (80)	0.426	19/29 (66)	48/55 (87)	**0.018**
**Anaemia**[2]	89/381 (23)	19/62 (31)	0.215	13/43 (30)	42/93 (45)	0.099
**BMI ≥ 30**	177/438 (40)	34 (48)	0.236	20 (43)	56 (54)	0.199
**Any Comorbidities**[3]	279 (63)	53 (75)	0.062	47 (100)	104 (100)	-

Data are in n (%) or n/N (%), in case of missing data. Only patients that presented hbA1C results were described. P-value <0,05 are highlighted.
1- "Corticosteroid use" refers to specific use at acute phase of disease. 2- Diagnostic based on the level of hemoglobin measured at first visit.3- Full list of comorbidities is described in table 01. 4- Newly diagnoses of DM.
