# Supplementary material for: Metabolic disorders and post-acute hospitalization in black/mixed-race patients with long COVID in Brazil: A cross-sectional analysis
Source: PLoS One. 2022 Oct 31;17(10):e0276771. doi: 10.1371/journal.pone.0276771 (PMC9621406; doi:10.1371/journal.pone.0276771)
Supplement: S4 Table — Data are in n/N(%). All reference values used to define laboratorial alteration are described in parenthesis. *Reference values for male and female, respectively. **N = 664. (PDF) [file pone.0276771.s006.pdf]

Supplementary Table 4 – Laboratorial blood analysis divided by time since acute disease.

	Time since symptom onset		
	1 |-- 2 months	2 |-- 3 months	>3 months
**Hemoglobin** (<14/12 g/dL)*	109/349 (31.2)	64/222 (28.8)	75/325 (23.1)
**White Blood Cell count per µl** (> 3500 or < 10500)	40/349 (11.5)	24/222 (10.8)	34/324 (10.5)
**Lymphocytes count per µl** (>700 or <4410)	22/347 (6.3)	20/213 (9.4)	28/315 (8.9)
**Platelet** (<14.000/dl or >450.000/dl)	7/348 (2.0)	13/222 (5.9)	5/324 (1.5)
**Urea** (<19/15 mg/dL or >43/36 mg/dL)*	57/337 (16.9)	42/221 (19)	65/315 (20.6)
**Creatinine** (>0.8/0.7mg/dL or <1.5/1.2 mg/dL)*	65/343 (19)	43/220 (19.5)	53/314 (16.9)
**Sodium** (<135 or >150 mEq/L)	17/340 (5.0)	3/211 (1.4)	14/313 (4.5)
**Potassium** (<3.5 or >5.0 mEq/L)	35/338 (10.4)	22/211 (10.4)	29/313 (9.3)
**Creatinine Phosphokinase** (>170/135 U/L)*	34/335 (10.1)	17/205 (8.3)	56/295 (19.0)
**C- Reactive Protein** (>5mg/L)	136/297 (45.8)	79/186 (42.5)	109/277 (39.4)
**Alanine Transaminase** (>72/52 U/L)*	29/345 (8.4)	18/214 (8.4)	13/310 (4.2)
**Aspartate Transaminase** (>59/36 U/L)*	22/346 (6.4)	9/215 (4.2)	10/311 (3.2)
**Albumin** (>3.2 g/dL or >4.8 g/dL)	13/288 (4.5)	13/162 (8.0)	10/247 (4.0)
**Total bilirubin** (>1.2 mg/dL)	9/304 (3.0)	9/184 (4.9)	7/278 (2.5)
**Glycated Hemoglobin****	*N=285*	*N=168*	*N=211*
<5.7%	89 (31.2)	68 (40.5)	81 (38.4)
5.7-6.4%	110 (38.6)	62 (36.9)	79 (37.4)
>6.4%	86 (30.2)	38(22.6)	51/ (24.2)

Data are in n/N(%). All reference values used to define laboratorial alteration are described in parenthesis.
**Reference values for male and female, respectively.*
***N=664*
