# Supplementary material for: Metabolic disorders and post-acute hospitalization in black/mixed-race patients with long COVID in Brazil: A cross-sectional analysis
Source: PLoS One. 2022 Oct 31;17(10):e0276771. doi: 10.1371/journal.pone.0276771 (PMC9621406; doi:10.1371/journal.pone.0276771)
Supplement: S5 Table — Data are n (%) or mean (±SD). (PDF) [file pone.0276771.s007.pdf]

Supplementary Table 5 - Characteristics of patients hospitalized one month or later after disease onset.

	**Mild acute disease (N=8)~3%**	**Moderate/Severe acute disease (N=43)~5%**
**Age, years**	42.5 (±5.2)	59.4 (±15.0)
**Female**	5 (62.5)	21 (48.8)
**ICU admission acute phase**	—	27 (43.0)
**Time after disease onset**		
**1-3 months**	6 (75.0)	32 (74.4)
**>3 months**	2 (25.0)	11 (25.6)
**Obesity (BMI≥30 kg/m^2)**	4 (50.0)	18 (41.8)
**Systemic Hypertension**	3 (37.5)	24 (55.8)
**Diabetes Mellitus**	1 (12.5)	15 (34.8)
**COPD**	0 (0.0)	5/42 (11.9)
**Cardiopathy**	0 (0.0)	7 (16.8)
**Bronchial asthma**	0 (0.0)	4 (10.0)
**Neoplasia**	0 (0.0)	3 (7.0)
**Chronic kidney disease**	0 (0.0)	4 (9.3)
**Cigarette smoking (former)**	1 (12.5)	19 (44.2)
**Cause of Hospitalization**		
**Infection**	2 (25.0)	11 (25.6)
**Pulmonary thromboembolism**	0 (0.0)	5 (11.6)
**Tracheal stenosis**	0 (0.0)	3 (7.0)
**Chest pain**	2 (25.5)	3 (7.0)
**Dyspnea**	1 (12.5)	11 (25.6)
**Others**	3 (37.5)	10 (23.3)

Data are n (%) or mean (±SD)
