# Supplementary material for: Metabolic disorders and post-acute hospitalization in black/mixed-race patients with long COVID in Brazil: A cross-sectional analysis
Source: PLoS One. 2022 Oct 31;17(10):e0276771. doi: 10.1371/journal.pone.0276771 (PMC9621406; doi:10.1371/journal.pone.0276771)
Supplement: S6 Table — Data are n (%) or mean (±SD). “N” represents the total sample of the subgroup. 1- P-value of analysis of each EuroQoL domains levels compared by sex and disease severity. (PDF) [file pone.0276771.s008.pdf]

Supplementary Table 6 – Descriptive EuroQol results separated by sex and disease severity at the acute phase.

	Mild			Moderate			Severe		
	**Male (N=75)**	**Female (N=224)**	**p value[1]**	**Male (N=123)**	**Female (N=139)**	**p value[1]**	**Male (N=202)**	**Female (N=166)**	**p value[1]**
**EuroQoL Global Score***	65.0 (±18.7)	62.0 (±18.1)	0.217	71.5 (±18.9)	64.8 (±20.2)	**0.007**	68.9 (±19.5)	68.2 (±18.9)	0.752
**Anxiety**	N=74	N=223	**0.003**	N=123	N=138	0.161	N=200	N=166	**0.008**
No anxiety/depression	27 (36.5)	43 (19.3)		53 (43.1)	39 (28.3)		67 (33.5)	33 (19.9)	
Slight anxiety/depression	20 (27)	49 (22)		23 (18.7)	32 (23.2)		46 (23.0)	43 (25.9)	
Moderate anxiety/depression	17 (23.0)	73 (32.7)		28 (22.8)	38 (27.5)		50 (25.0)	36 (21.7)	
Severe anxiety/depression	9 (12.2)	42 (18.8)		16 (13.0)	21 (15.2)		28 (14.0)	37 (22.3)	
Extreme anxiety/depression	1 (1.4)	16 (7.2)		3 (2.4)	8 (5.8)		9 (4.5)	17 (10.2)	
**Mobility**	N=75	N=224	0.052	N=123	N=139	**0.005**	N=202	N=166	0.224
No problems with walking around	63 (84.0)	161 (71.9)		95 (77.2)	84 (60.4)		125 (61.9)	90 (54.2)	
Slight problems with walking around	10 (13.3)	29 (12.9)		9 (7.3)	24 (17.3)		33 (16.3)	31 (18.7)	
Moderate problems with walking around	1 (1.3)	26 (11.6)		11 (8.9)	20 (14.4)		25 (12.4)	32 (19.3)	
Severe problems with walking around	1 (1.3)	5 (2.2)		5 (4.1)	11 (7.9)		11 (5.4)	10 (6)	
Unable to walk around	0 (0.0)	3 (1.3)		3 (2.4)	0 (0.0)		8 (4.0)	3 (1.8)	

**Pain/Discomfort**	N=74	N=224		N=123	N=139	**<0.001**	N=198	N=166	**0.003**
No pain/discomfort	31 (41.9)	43 (19.2)		59 (48.0)	35 (25.2)		89 (44.9)	49 (29.5)	
Slight pain/discomfort	21 (28.4)	52 (23.2)		33 (26.8)	32 (23)		48 (24.2)	42 (25.3)	
Moderate pain/discomfort	16 (21.6)	91 (40.6)		24 (19.5)	43 (30.9)		32 (16.2)	49 (29.5)	
Severe pain/discomfort	6 (8.1)	32 (14.3)		7 (5.7)	26 (18.7)		22 (11.1)	23 (13.9)	
Extreme pain/discomfort	(0.0)	6 (2.7)		(0.0)	3 (2.2)		7 (3.5)	3 (1.8)	
**Self Care**	N=75	N=224	0.136	N=122	N=139	0.074	N=202	N=166	0.188
No problems with washing or dressing	70 (93.3)	182 (81.3)		107 (87.7)	107 (77.0)		160 (79.2)	120 (72.3)	
Slight problems with washing or dressing	2 (2.7)	20 (8.9)		7 (5.7)	13 (9.4)		13 (6.4)	19 (11.4)	
Moderate problems with washing or dressing	2 (2.7)	14 (6.3)		4 (3.3)	11 (7.9)		14 (6.9)	19 (11.4)	
Severe problems with washing or dressing	1 (1.3)	5 (2.2)		1 (0.8)	6 (4.3)		8 (4.0)	5 (3.0)	
Unable to wash or dress	(0.0)	3 (1.3)		3 (2.5)	2 (1.4)		7 (3.5)	3 (1.8)	
**Usual Activities**	N=75	N=223	0.103	N=123	N=138	**<0.001**	N=201	N=166	**0.031**
No problems with usual activities	42 (56.0)	96 (43.0)		71 (57.7)	47 (34.1)		99 (49.3)	61 (36.7)	
Slight problems with usual activities	13 (17.3)	49 (22.0)		21 (17.1)	38 (27.5)		37 (18.4)	41 (24.7)	
Moderate problems with usual activities	15 (20.0)	59 (26.5)		18 (14.6)	38 (27.5)		39 (19.4)	33 (19.9)	

Severe problems with usual activities	5 (6.7)	10 (4.5)	8 (6.5)	14 (10.1)	12 (6.0)	18 (10.8)
Unable to do usual activities	(0.0)	9 (4.0)	5 (4.1)	1 (0.7)	14 (7.0)	13 (7.8)

Data are n (%) or mean (±SD). “N” represents the total sample of the subgroup.
1- P-value of analysis of each EuroQoL domains levels compared by sex and disease severity.
