# Supplementary material for: Metabolic disorders and post-acute hospitalization in black/mixed-race patients with long COVID in Brazil: A cross-sectional analysis
Source: PLoS One. 2022 Oct 31;17(10):e0276771. doi: 10.1371/journal.pone.0276771 (PMC9621406; doi:10.1371/journal.pone.0276771)
Supplement: S7 Table — (PDF) [file pone.0276771.s009.pdf]

Supplementary Table 7 – Ordinal Logistic regression for EuroQoL domains

	Mobility			Self-Care			Usual Activities			Anxiety/depression			Pain/discomfort		
	Odds Ratio	95% CI	p value	Odd's Ratio	95% CI	p value	Odd's Ratio	95% CI	p value	Odd's Ratio	95% CI	p value	Odd's Ratio	95% CI	p value
**Fatigue**	2.23	1.60 to 3.14	<0.001	2.80	1.81 to 4.44	<0.001	2.93	2.19 to 3.95	<0.001	2.36	1.78 to 3.11	<0.001	2.53	1.94 to 3.32	<0.001
**Chest pain**	1.28	0.95 to 1.73	0.10	1.68	1.16 to 2. 44	0.006	1.46	1.12 to 1.89	0.005	2.82	2.17 to 3.68	<0.001	1.24	0.96 to 1.59	0.10
**Dyspnea**	1.32	0.96 to 1.82	0.090	1.54	1.03 to 2.35	0.038	1.74	1.31 to 2.31	<0.001	1.15	0.87 to 1.50	0.33	1.36	1.05 to 1.77	0.020
**Severe Acute illness**	2.23	1.57 to 3.21	<0.001	1.87	1.21 to 2.90	0.005	1.44	1.05 to 1.96	0.023	0.88	0.65 to 1.19	0.41	1.23	0.91 to 1.65	0.18
**Moderate Acute illness**	1.44	0.98 to 2.13	0.064	1.21	0.75 to 1.94	0.44	1.17	0.84 to 1.62	0.36	0.87	0.63 to 1.20	0.40	0.83	0.61 to 1.14	0.25
**BMI- kg/m^2**	1.00	0.97 to 1.03	>0.99	0.98	0.95 to 1.02	0.28	0.98	0.96 to 1.01	0.17	1.03	1.00 to 1.05	0.030	1.01	0.98 to 1.03	0.55
**Sex (Female)**	1.47	1.09 to 2.00	0.011	1.58	1.09 to 2.31	0.018	1.43	1.10 to 1.87	0.009	1.87	1.44 to 2.43	<0.001	1.66	1.29 to 2.13	<0.001
**Age, years**	1.04	1.03 to 1.05	<0.001	1.04	1.03 to 1.06	<0.001	1.02	1.01 to 1.03	0.001	1.01	1.00 to 1.02	0.013	0.99	0.98 to 1.00	0.077
**Any comorbidity**	1.02	0.70 to 1.50	0.90	1.51	0.93 to 2.48	0.10	1.32	0.95 to 1.83	0.095	0.98	0.71 to 1.34	0.880	1.45	1.06 to 1.99	0.019
