# Supplementary material for: Metabolic disorders and post-acute hospitalization in black/mixed-race patients with long COVID in Brazil: A cross-sectional analysis
Source: PLoS One. 2022 Oct 31;17(10):e0276771. doi: 10.1371/journal.pone.0276771 (PMC9621406; doi:10.1371/journal.pone.0276771)
Supplement: S8 Table — (PDF) [file pone.0276771.s010.pdf]

Supplementary Table 8 – Multiple linear regression for EuroQoL Global Score

	**Global**		
	Beta (Estimate)	95% CI	p value
**Fatigue**	-9.8	-12 to -7.1	<0.001
**Chest pain**	-7.3	-9.8 to -4.7	<0.001
**Dyspnea**	-5.4	-8.0 to -2.8	<0.001
**Severe**	4.3	1.3 to 7.3	0.005
**Moderate**	4.0	0.8 to 7.1	0.014
**BMI- kg/m^2**	-0.04	-0.27 to 0.20	0.77
**Sex (Female)**	-0.34	-2.9 to 2.2	0.79
**Age, years**	-0.16	-0.26 to -0.05	0.002
**Any comorbidity**	-1.6	-4.7 to 1.5	0.31
