# Supplementary material for: Metabolic disorders and post-acute hospitalization in black/mixed-race patients with long COVID in Brazil: A cross-sectional analysis
Source: PLoS One. 2022 Oct 31;17(10):e0276771. doi: 10.1371/journal.pone.0276771 (PMC9621406; doi:10.1371/journal.pone.0276771)
Supplement: Attachment — Submitted filename: Response to Reviewers.pdf [file pone.0276771.s012.pdf]

Ministério da Saúde

FIOCRUZ
Fundação Oswaldo Cruz
Centro de Pesquisas Gonçalo Moniz

September 06h, 2022

Dear Lucien Peroni Gualdi,
Academic Editor
PLOS ONE

Thank you for sending us the reviewers' comments on our manuscript "Metabolic disorders and re-hospitalization in black/mixed-race patients with long COVID in Brazil". We are pleased that the referees and the editors found our work interesting and are grateful for their constructive comments for improving the paper.

We have carefully revised the manuscript, and a detailed point-by-point response to the editor and referees is provided below, with the changes in the manuscript text highlighted in a separate document.

We look forward to hearing a positive response to this manuscript.

Best regards,

Viviane Sampaio Boaventura

PhD Fiocruz, Bahia, Brazil

PONE-D-22-10158

Editor (E) Comment

E*: Please ensure that your manuscript meets PLOS ONE's style requirements, including those for file naming.*

Answer (A): All manuscript formatting has been updated to reflect the PLOS style, including file naming

E: *Please state what role the funders took in the study. If the funders had no role, please state: ""The funders had no role in study design, data collection and analysis, decision to publish, or preparation of the manuscript."" If this statement is not correct you must amend it as needed.*

A: The funders had no role in study design, data collection, and analysis, decision to publish, or preparation of the manuscript.

The final version of the text is: "MABF received a Scientific Initiation Scholarship - PIBIC from "Conselho Nacional de Desenvolvimento Científico e Tecnológico" (CNPq) -Brazil; T.C.-S. is a PhD student at the Post-Graduation Program in Health Sciences-UFBA, which is supported by the "Coordenação de Aperfeiçoamento de Pessoal de Nível Superior"-Brasil, finance code 001. A C. reports consulting fees from "Fundação Maria Emilia Freire". MB-N and VSB are research fellows from CNPq. The funders had no role in study design, data collection and analysis, decision to publish, or preparation of the manuscript."

E*: In your Data Availability statement, you have not specified where the minimal data set underlying the results described in your manuscript can be found [...]. We note that you have stated that you will provide repository information for your data at acceptance. Should your*

manuscript be accepted for publication, we will hold it until you provide the relevant accession numbers or DOIs necessary to access your data.

A.: We provided a document with de-identified data, along with a codebook describing the tabulation and codification of each variable. The document was attached as a supplementary file in the "Supporting Information files" within the PLOS Platform.

E*: If you wish to make changes to your Data Availability statement, please describe these changes in your cover letter and we will update your Data Availability statement to reflect the information you provide.*

A: The updated version of the Data Availability statement will be: "De-identified minimal data set can be found as supplementary material."

E*: One of the noted authors is a group or consortium [CPC study group]. In addition to naming the author group, please list the individual authors and affiliations within this group in the acknowledgments section of your manuscript. Please also indicate clearly a lead author for this group along with a contact email address.*

A: The complete list of authors and affiliations of the "CPC study group" was updated at the acknowledgment sections. The lead author and the email address were provided.

Answers to Reviewer #1 comments:

Thank you for the opportunity to review the manuscript "Metabolic disorders and re-hospitalization in black/mixed-race patients with long COVID in Brazil". This study describes a cohort of adults seeking care in a post COVID care clinic in Brazil between August 2020 and September 2021. Outcomes evaluated included hospital readmission, HbA1c levels, long COVID symptoms and validated quality of life survey results. The cohort included patients who were outpatients as well as inpatients for their acute COVID infection. This study provides insights into long COVID in an understudied population (LMIC, black and mixed race), with findings that suggest that there is little difference in presentation by race/ethnicity and provides interesting laboratory data about this important population. However, there are several limitations worth noting that dampen my overall enthusiasm for this manuscript.

A: We thank the reviewer for the criticism. We have made several modifications in the MS in order to address these concerns, as detailed below.

R#1: Q1. The population evaluated is a mix of inpatients, all of whom are referred to the clinic, and outpatients, who comprise individuals are seeking medical attention due to their symptoms, preventing any real conclusions to be drawn from the comparisons of mild and moderate/severe disease, or the frequency of symptoms in this population.

A: We agree with reviewer 1. Therefore, we did not compare groups with different degrees of severity. Rather, we aimed to evaluate the relative difference between women and men with the same disease severity during acute infection. In this context, we observed that several long COVID symptoms were more frequently reported by women compared to men across different acute disease severity.
We also reinforced this limitation in the discussion section:
"Second, our current study was unable to access the prevalence of COVID in the population, nor the differences in clinical presentation of long COVID across groups of mild, moderate, and severe acute infection. These limitations are related to the strategy of the recruitment."

.

R#1: Q2. The population is biased based on the above reasons, but also may over-represent patients with respiratory complaints.

A: We have rewritten this limitation statement in the discussion section:

"The CPC resides in a reference hospital for respiratory diseases, and cases with respiratory complaints may have been preferentially referred to this health unit. However, it´s important to highlight that the CPC was the reference unit for long COVID in the Bahia State, which may have attenuated the selection bias. Indeed, several non-respiratory post-COVID symptoms were detected in our study, as in other reports from the literature."

R#1: Q3. The absence of a control group prevents true understanding of which symptoms and QoL measures may be a direct effect of SARS CoV2 infection, versus the situational context of the pandemic.

A: We agree with the reviewer. We have reinforced this limitation in the discussion section:

"Third, we were also unable to conclude if the abnormal laboratory exams/ disturbance in QoL were a direct effect of COVID-19 or due to previous conditions/stress related to the COVID-19 pandemic. Further cohort studies including baseline exams/EuroQoL measures should be performed to evaluate the causality."

R#1: Q4. The assessment of readmissions is incomplete and non-contributory- as it is not known whether these readmissions were related to long COVID symptoms or underlying conditions.

A: Unfortunately, we were unable to deeply evaluate the post-acute hospitalization due to limited data on discharge summaries. Neither was possible to characterize the causal relationship between hospitalization and COVID-19. Nevertheless, we believe that the high frequency of readmissions of moderate/severe cases (5%) and hospitalization of mild cases (3%) short-term after acute COVID-19 should be described in the study considering its potential impact on the public health system. We have stated this limitation in the discussion section: "…due to limited discharge summary data, we cannot conclude if post-acute hospitalization stems from long COVID symptomatology".

R#1: Q5. There was no standardized follow up period, making the laboratory value interpretation difficult, as well as the reported symptoms. Some stratifying by time period after acute infection may therefore be helpful.

A: Indeed, a limitation of our project was not obtaining a standardized follow-up period, as stated in the limitation paragraph. Nevertheless, we were able to categorize patients according to time since symptom onset and then assess the frequency of abnormal tests in our sample. As stated below, CPR, hemoglobin, and HbA1c remain the most frequent laboratory alteration regardless of time since symptom onset. This table was included as supplementary material (S4 table) and the information added to result section.

	**TIME SINCE SYMPTOM ONSET**		
	1 |-- 2 months	2 |-- 3 months	>3 months
**Hemoglobin** (<14/12 g/dL)*	109/349 (31.2)	64/222 (28.8)	75/325 (23.1)
**White Blood Cell count per µl** (> 3500 or <10500)	40/349 (11.5)	24/222 (10.8)	34/324 (10.5)
**Lymphocytes count per µl** (>700 or <4410)	22/347 (6.3)	20/213 (9.4)	28/315 (8.9)
**Platelet** (<14.000/dl or >450.000/dl)	7/348 (2.0)	13/222 (5.9)	5/324 (1.5)
**Urea** (<19/15 mg/dL or >43/36 mg/dL)*	57/337 (16.9)	42/221 (19)	65/315 (20.6)
**Creatinine** (>0.8/0.7mg/dL or <1.5/1.2 mg/dL)*	65/343 (19)	43/220 (19.5)	53/314 (16.9)
**Sodium** (<135 or >150 mEq/L)	17/340 (5.0)	3/211 (1.4)	14/313 (4.5)
**Potassium** (<3.5 or >5.0 mEq/L)	35/338 (10.4)	22/211 (10.4)	29/313 (9.3)
**Creatine Phosphokinase** (>170/135 U/L)*	34/335 (10.1)	17/205 (8.3)	56/295 (19.0)
**C-Protein Reactive** (>5mg/L)	136/297 (45.8)	79/186 (42.5)	109/277 (39.4)
**Alanine Transaminase** (>72/52 U/L)*	29/345 (8.4)	18/214 (8.4)	13/310 (4.2)
**Aspartate Transaminase** (>59/36 U/L)*	22/346 (6.4)	9/215 (4.2)	10/311 (3.2)
**Albumin** (>3.2 g/dL or >4.8 g/dL)	13/288 (4.5)	13/162 (8.0)	10/247 (4.0)
**Total bilirubin** (>1.2 mg/dL)	9/304 (3.0)	9/184 (4.9)	7/278 (2.5)
**Glycated Hemoglobin****	N=285	N=168	N=211
. <5.7%	89 (31.2)	68 (40.5)	81 (38.4)
5.7-6.4%	110 (38.6)	62 (36.9)	79 (37.4)
>6.4%	86 (30.2)	38(22.6)	51/ (24.2)

Data are in n/N(%). All reference values used to define laboratorial alteration are described after the exam.
**Reference values for male and female, respectively.*
***N=664*

R#1: Q6. Interpretation of HbA1c is also difficult given the summary data doesn't take into account those with existing diabetes, nor the time after acute infection, or other factors that

may influence these results (such as Hemoglobin, corticosteroid use and patient risk factors) and there are no baseline data available for comparison.

A: We agree that a number of clinical factors could falsely influence HbA1C levels. Therefore, we have included a supplementary table with the potential confounders cited above (S3 Table).

Use of corticosteroids, anemia and obesity/comorbidities were similar among Long COVID cases with and without high levels of HbA1c. This information was added in the result section.

	PREVIOUS DIAGNOSES OF DM					
	No			Yes		
**HbA1c**	<6.4% N=441	>6.4% N=71	P-Value	<6.4% N=47	>6.4% N=104	P-Value
**Time since disease onset**	2.9 (±1.9)	2.9 (±1.9)	0.697	3.2 (±2.0)	2.6 (±1.8)	0.352
**Corticosteroid use[1]**	210/284 (74)	35/44 (80)	0.426	19/29 (66)	48/55 (87)	**0.018**
**Anaemia[2]**	89/381 (23)	19/62 (31)	0.215	13/43 (30)	42/93 (45)	0.099
**BMI ≥ 30**	177/438 (40)	34 (48)	0.236	20 (43)	56 (54)	0.199
**Any Comorbidities[3]**	279 (63)	53 (75)	0.062	47 (100)	104 (100)	-

Data are in n (%) or n/N (%), in case of missing data. Only patients that presented hbA1C results were described.
1- “Corticosteroid use” refers to specific use at acute phase of disease. 2- Diagnostic based on the level of hemoglobin measured at first visit.3- Full list of comorbidities is described in table 01. 4- Newly diagnoses of DM.

Minor comments:

a) *Introduction*

R#1: Q7. Please state the objectives in the final paragraph more clearly, e.g. That the main objectives were to describe the symptoms associated with long COVID and include a description of which complications were being evaluated.

A: The objectives were stated as suggested: “We aimed to describe persistent and late-onset symptoms associated with long COVID, and complications temporarily related, such as hospital readmission and metabolic disorders. We also evaluated the quality of life (QoL) of patients presenting Long COVID symptoms.”

b) *Methods*

R#1: Q8 A description of which patients are being seen at CPC would be helpful in the methods, that all inpatients are referred, but only those with persistent symptoms with outpatient COVID are being self-referred/referred in.

A: We have added the sentence as suggested. The complete text now reads: "Patients were recruited by invitation upon discharge from local COVID-19 reference hospitals or came to CPC as outpatients (advertisements were circulated via conventional and social media"). All inpatients were referred, but only those with persistent symptoms with outpatient COVID were being self-referred/referred in."

R#1: Q9 Would recommend excluding patients with clinical features of viral pneumonia, since this is not discriminative enough for COVID-19 infection.

A: We used clinical feature of viral pneumonia as an inclusion criterion because: 1. confirmatory tests were scarce in the first year of the pandemic; 2. In addition to sign/symptoms of viral pneumonia, computed tomographic lung scan compatible with COVID-19 was required, as stated in the Methods section. 3. Recruitment occurred between August 2020 and September 2021, when other respiratory viruses corresponded to less than 1% of all viral pneumonia detected by the national surveillance system (Link: *https://www.gov.br/saude/pt-br/centrais-de-conteudo/publicacoes/boletins/epidemiologicos/covid-19/2021/boletim_epidemiologico_covid_73-4-final3ago.pdf/@@download/file/Boletim_epidemiologico_COVID_73%20(4)FINAL3ago.pdf accessed on August 31, 2022*). Additionally, only 16 out of 1164 (1,4%) patients were included by clinical/radiologic criteria, minimizing any impact of this cases on results.

R#1: Q10 Statistical analyses: ordinal logistic regression analyses did not account for underlying medical condition, which is another important covariate to consider.

A: We have included comorbidity to the ordinal logistic regression models. All tables and figures related were updated

c) Results:

R#1: Q11. Suspected COVID-19 included those with a CT showing viral infection, but 16 patients were excluded due to no confirmatory diagnosis of COVID-19 which is confusing and doesn't match the inclusion/exclusion criteria from the methods.

A: We thank the reviewer for pointing out this mistake. These 16 cases did not present the criteria to be included in the study and, therefore, were removed from the description/Flowchart.

.

R#1: Q12. Residual symptoms are usually initiated at the acute stage of disease"- residual by definition implies that the same symptoms were present during acute illness, so this statement is better switched to "new symptoms including chest pain, myalgia and fatigue were reported after recovery from acute illness". However, this may be subject to recall bias.

A: We have modified the sentence to: "Long COVID complaints usually initiated at the acute stage of disease, persisting as residual symptoms. Some patients with long COVID reported *new symptoms after recovery from acute illness including chest pain, myalgia, and fatigue* (S1 Table and S1 Fig)." (Page 07, line 158)

R#1: Q13. Table 2- there are many analyses included here, increasing the likelihood of chance findings, and a test for multiple comparisons would be beneficial.

A: We have performed the P-value adjustment for multiple comparisons in Table 02.

R#1: Q14. The HbA1c data would be helpful to be represented as a scatter plot, with the y axis being the HbA1c value, the x axis being time since acute infection in weeks/months, and two different sets of data including 1) those with a history of diabetes and 2) those values among patients without a history of diabetes.

A: We thank the reviewer's suggestion. A chart with the HbA1c data across the time was presented in S2 Fig. Levels of HbA1c varied independently of the time after disease onset for all groups (previous diagnosis of DM, no history of DM and high HbA1c, and no history of DM and low HbA1c). This information was added on page 09 line 174.

R#1: Q15. Table 3. Suggest stratifying laboratory findings based on outpatient, hospitalized and ICU admission for acute COVID, which may be more interesting to readers.

A: We added three columns to table 03, stratifying the data according to acute disease severity. No comparative analysis was performed considering baseline differences between groups.

R#1: Q16 "Late hospitalization" is a confusing term, and the statement about 8 patients presenting with mild COVID-19 warrants clarification. Isn't mild defined here as outpatient illness? And re-testing within 90 days could represent viral shedding rather than new infection.

A: We have changed the term "late hospitalization" to "post-acute hospitalization".

Regarding the contradiction of hospitalization of mild cases, these eight patients were hospitalized after 30 days of disease onset, due to infection, chest pain, and dyspnea, among other reasons. No re-test for COVID-19 was performed and we cannot link the post-acute hospitalization to the SARS-CoV-2 infection, as stated in the limitation.

To avoid misunderstanding, we have modified the term mild patient to "mild acute disease" when referring to post-acute hospitalization.

R#1: Q 17 Were there any changes in presentation, symptom reporting by calendar time?

A: We thank the reviewer for this suggestion. Clinical presentation of Long COVID varied with calendar time. Olfactory and gustatory dysfunctions were more frequently detected during the circulation of ancestral strain compared to the period of gamma/delta variant (S2 Table). This information was added in the MS:

	**Ancestral N=736**	**Gamma-Variant N=249**
**Female Sex**	440/736 (59.8)	117/249 (47.0)
**Age**	52.1 (±14.2)	52.1 (±11.9)
**BMI**	29.7 (6.2)	30.3 (6.3)
**Any Comorbidities**	527/736 (71.6)	187/248 (75.4)
**Hospitalization**	443/736 (60.2)	236/249 (94.8)
ICU	236/443 (53.3)	167/236 (70.7)
**Headache**	243/685 (35.5)	83/248 (33.5)
**Cough**	269/735 (36.6)	106/249 (42.6)
**Dyspnea**	486/736 (66.0)	165/249 (66.3)
**Myalgia**	273/735 (37.1)	106/249 (42.6)
**Fatigue**	466/735 (63.4)	140/249 (56.2)
**Chest Pain**	337/734 (45.9)	95/249 (38.2)
**Appetite Loss**	121/734 (16.5)	33/237 (13.9)
**Dysphagia**	36/734 (4.9)	9/233 (3.9)
**Dysphonia**	39/734 (5.3)	12/234 (5.1)
**Gustatory disfunction**	124/622 (19.9)	17/233 (7.3)
**Smell Loss**	130/620 (21.0)	23/232 (9.9)
**Motor limitation**	107/619 (17.3)	52/235 (22.1)
**Hair Loss**	65/181 (35.9)	97/238 (40.8)
**Dizziness**	70/181 (38.7)	71/244 (29.1)
**Insomnia**	96/181 (53.0)	122/245 (49.8)
**Memory loss**	100/182 (54.9)	136/244 (55.7)

R#1: Q18 Figure 3- EuroQoL plot is difficult to follow, suggest some re-organizing with a figure legend description.

A: We reorganized Figure 3 legend as suggested.

“Fig 3 - Forest plot of ordinal logistic regression for each domain of EuroQoL (Mobily, Self-care, Usual Activities, Anxiety/Depression, and Pain/discomfort). Each symbol represents the odds ratio with the corresponding 95% confidence interval of presenting alteration (≥2 points) in each domain of EuroQoL separated by predictor variables. Significant results (p<0.05) are highlighted with a red line. Descriptive results are displayed in the supplementary material.”

d) Discussion

R#1: Q19 *In the main findings paragraph, would also include that there was no change by race and ethnicity.*

A: The first paragraph of the discussion was altered to include this information: “Our analysis showed that long COVID mainly affected women, causing dyspnea, fatigue, and chest pain regardless of COVID-19 severity, but no change was observed by race and ethnicity.”

R#1: Q20 Discussion, paragraph 2 - Huang report – “reported 58…” – need denominator to be included in this sentence.

A: Denominator was included in the sentence: “…reported 58 out of 1526 patients presenting newly diagnosed DM after discharge” (Page 11, Line 223)

R#1: Q21 Page 13, line 16. Do not understand the term “chronic health system deficiencies” – does this mean “underlying medical conditions”?

A: To avoid misunderstanding, we have changed the term "chronic health system deficiencies" to "long-standing deficiencies in the Brazilian public health system".

R#1: Q22. Limitations- would include additional limitations outline above.

A: We are grateful for all above points discussed by reviewer 01. Methods and limitations were significantly restructured in the revised version of the manuscript.

R#1: Q23 Additional comments: Several typographical errors are noted throughout, e.g. Symptoms (line 10, abstract), c-protein reactive instead of C- reactive protein (results).

A: Manuscript was carefully checked for typographical errors.

Reviewer #2 comments:

Many thanks for asking me to review this manuscript entitled "Metabolic disorders and re-hospitalization in black/mixed-race patients with long COVID in Brazil "

R#2: Q1. The approach used by authors is comprehensive and could be interesting within a process of quality improvement in the management of the post-COVID resulting from mild to severe COVID-19 which is why I enjoyed reading this manuscript.

A: We Thank the reviewer for the positive comment.

R#2: Q2. 1/ Title is clear but should display the study's design.

A: We have changed the manuscript title to "Metabolic disorders and re-hospitalization in black/mixed-race patients with long COVID in Brazil: a cross-sectional analysis"

R#2: Q3/ Abstract. Authors provided along with an informative and balanced summary of what was done and what was found. Authors may just reorganize abstract in a structured manner.

A: The abstract was reorganized in subtopics as suggested.

R#2: Q4 Second sentence is an only very long methods-related phrase!

A: We agree with the reviewer. We have modified this part of the abstract.

R#2: Q5 The aim of the study should be individualized.

A: We included a sentence with the aim of the study: "This study aimed to evaluate long COVID symptomatology, complications (hospital readmission and metabolic disorders) and main clinical features that impact Quality of Life (QoL). "

R#2: Q6 Results should be presented in a uniform manner, n (%) (The example of HbA1c with no %).

A: The result section of the abstract was modified as suggested.

3/ Introduction

R#2: Q7 Authors explained well the scientific background and rationale for the investigation being reported and stated specific objectives.

A: We thank the reviewer for the feedback.

R#2: Q8 Methods Authors presented key elements of study design early in the section. They described the setting, locations, and relevant dates, including periods of recruitment and data collection. They gave the eligibility criteria, and the sources and methods of selection of participants. They clearly addressed needed definitions. They explained how the study size was arrived at. They described all statistical methods, including those used to control for confounding. Authors are encouraged to describe more their efforts to address potential sources of bias and explain how missing data were addressed in this section.

A: We thank the reviewer for the comments. Missing data were present in some variables and were assumed to be missing completely at random. No imputation was realized and all variables with missing data were described as n/N (%). Multivariate regression analysis does not contemplate these cases. To prevent bias due to recruitment differences, all bivariate analyses were realized separately in each group of disease severity and performed P-value adjustment for multiple comparisons. The methods and limitation sections were carefully revised to describe our effort to address potential bias.

5/ Results

R#2: Q9 The flow diagram is appreciated. Table 2, I suggest authors adding a column with non-separated values.

A: We thank the reviewer for the suggestion. An overall column was added to table 02.

R#2: Q10 All results are given and displayed within comprehensive and systemic tables and figures. Patients characteristics, information on exposures and potential confounders are given. They indicated number of participants with missing data for each variable of interest.

A: We thank the reviewer for the comments.

R#2: Q11/ Discussion

Authors summarized key results with reference to study objectives. Authors addressed cautious overall interpretation of results considering objectives, limitations, multiplicity of analyses, results from similar studies, and other relevant evidence according to Brazil population. Authors are suggested to start with dyspnea, then glucose intolerance, then QoL as the order adopted in the abstract and results sections; unless they think that glucose intolerance is more important to start with.

A: Thank you again for the positive comments. The choose to maintain the order of the subjects in discussion section, considering the relevance of the data about abnormal HbA1c levels after COVID-19 in patients without history of diabetes.

R#2: Q12 Limitations were well discussed.

A: We thank the reviewer for the feedback.

Reviewer #3 comments:

Ana Paula Andrade Barreto et al did a cross-sectional analysis with a detailed clinical picture for people with long COVID or post-COVID-19 condition (WHO definition). A total of 1164 (88% black/mixed-race) were evaluated 2.3 [IQR=1.6-3.7] months after mild (30.2%), moderate (29.0%) or severe (40.8%) acute illness. They concluded that in this majority-black/mixed-race population in Brazil, long COVID was associated with re-hospitalization, newly diagnosed diabetes mellitus, and decreased quality of life, particularly in women and

regardless of disease severity of acute infection. These findings with rigorous statistical analysis in a large-size sample validate previous published evidence of mostly black and mixed-race individuals with long COVID. Given that COVID-19 now affects millions of people worldwide, long COVID has become a meaningful public health concern and may exacerbate chronic health system deficiencies in low-middle income countries. Investigation of long COVID with valuable clinical manifestation provides important implications for health care system and development of effective treatment for this complicated syndrome.

A: Thank you for the comments

Suggestion and Question:

R#3: Q1. Some longitudinal analysis on recovery from long COVID in this group of people will make this manuscript more valuable and informative, but it is not necessary for publication.

A: We agree that a longitudinal analysis would greatly increase the value of this study. However, these results are not available by now.

*R#3: Q*2 In Supplementary Table 3, please clarify the P value following N number for which group of data.

A: The P-value following "N" number represents the analysis between the values of each EuroQoL domain, stratified by sex and disease severity.

For example, the P-value of 0.003 (line 2) refers to the analysis of mobility severity between men and women for mild cases. The N value only represents the total number of patients in the sample as an effort to report all missing cases.

We added the above explanation to S6 Table legend to avoid further misunderstanding.
